# Recombination Pathways in Green InGaN/GaN Multiple Quantum Wells

**DOI:** 10.1186/s11671-017-1922-2

**Published:** 2017-02-21

**Authors:** Tao Lin, Hao Chung Kuo, Xiao Dong Jiang, Zhe Chuan Feng

**Affiliations:** 1 Laboratory of Optoelectronic Materials & Detection Technology, Guangxi Key Laboratory for Relativistic Astrophysics, School of Physical Science & Technology, Guangxi University, Nanning, 530004 China; 2 Department of Photonics & Institute of Electro-Optical Engineering, National Chiao Tung University, Hsinchu City, 30010 Taiwan

**Keywords:** Light-emitting diodes, Photoluminescence, Exciton localization

## Abstract

This paper reports the transient photoluminescence (PL) properties of an InGaN/GaN multiple quantum well (MQW) light-emitting diode (LED) with green emission. Recombination of localized excitons was proved to be the main microscopic mechanism of green emission in the sample. The PL dynamics were ascribed to two pathways of the exciton recombination, corresponding to the fast decay and the slow decay, respectively. The origins of slow decay and fast decay were assigned to local compositional fluctuations of indium and thickness variations of InGaN layers, respectively. Furthermore, the contributions of two decay pathways to the green PL were found to vary at different emission photon energy. The fraction of fast decay pathway decreased with decreasing photon energy. The slow radiative PL from deep localized exciton recombination suffered less suppression from non-radiative delocalization process, for the higher requested activation energy. All these results supported a clear microscopy mechanism of excitation-emission process of the green MQW LED structure.

## Background

InGaN/GaN multiple quantum well (MQW) light-emitting diodes (LEDs) have attracted much attention for the potential application in next generation solid-state lighting. However, the internal quantum efficiency (IQE) of the green emission from MQW structure has suffered from a dramatical decrease compared with that from blue emission [1–4]. This drawback strongly hindered their full-color applications. This “green gap” has been attributed to high dislocation density that resulted from the large lattice mismatch between InGaN and GaN, which was deteriorated by adding extra indium component for narrowing down the well bandgaps [5–9]. For a typical blue MQW LED, exciton localization effect has been proposed to improve the IQE, which was related to several structural imperfections [10–13], such as compositional fluctuations of indium within InGaN wells [14, 15], formation of dot-like In-rich clusters [16–18] and well-width fluctuations in the activated layers [19], all of which were dependent on indium fractions in the wells. Furthermore, photoluminescence (PL) decays were found deviating from single-exponential decay, which indicated multiple exciton localization origins simultaneously functioning in MQW structures. Hence, it is reasonable to assume that the drop of IQE is related to changes of the nature of these structural imperfections and is necessary to analyze PL dynamic properties and recombination pathways in detail, especially that for the green emission band, which is broad and complicated. To date, although many efforts have been performed to analyze the dynamic properties of green emission point by point to each wavelength involved in emission band, giving the conclusion that the whole emission band may contributed by exciton localization [20, 21], few attention has been paid to the existence of multiple PL pathways for green emission.

In this work, steady-state (SS) PL spectra and time-resolved (TR) PL spectra of green emission from an InGaN/GaN MQW LED were measured to analyze the luminescence properties. Also, the temperature-dependent and emission photon energy-dependent PL efficiencies and PL lifetimes were measured to achieve the activation energy and dynamic properties of green emission at different emission photon energy. The fast decay and slow decay were extracted simultaneously from time-resolved PL (TRPL) for the purpose of evaluating different PL pathways contributing to the green emission. We found that the slow PL process ascribable to local compositional fluctuations of indium had better resistance to the suppression of non-radiative recombination than the fast one ascribable to well thickness variation. This may guide the device fabrications and improve the device efficiencies in the future.

## Methods

**Fig. 1:**
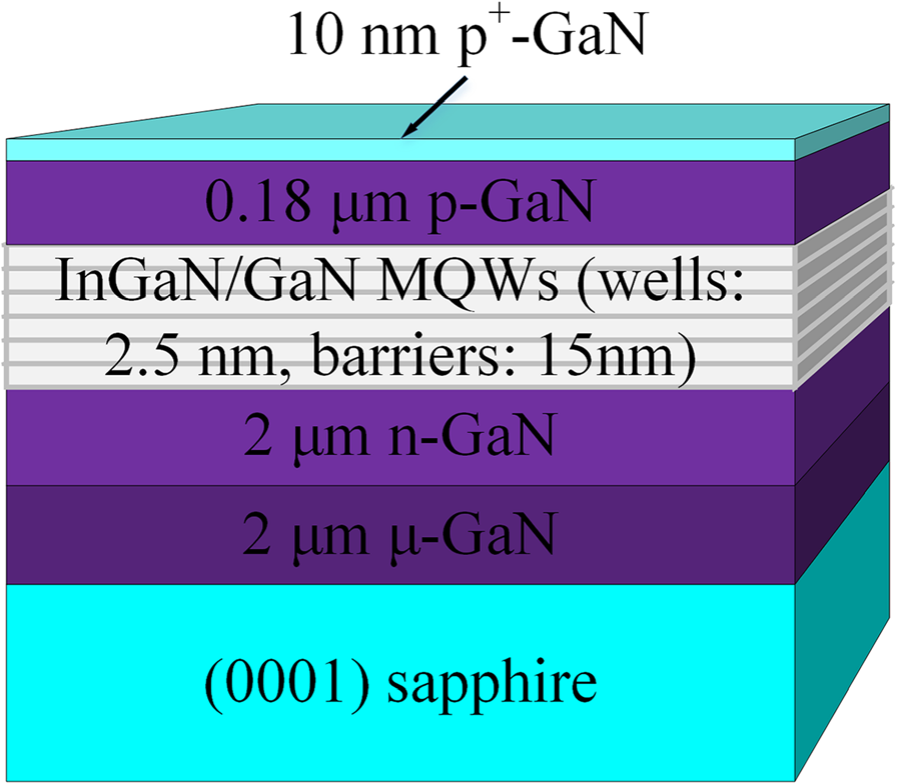
Structure of the green MQW LED sample

As shown in the schematic of Fig. 1, the epitaxial growth of InGaN/GaN MQWs were performed by metal organic chemical vapor deposition: 2 μm μ GaN buffer layer was grown on *c*-plane (0001) sapphire at 520 °C, followed by 2 μm n-type GaN grown at 1020 °C. Then six periods of InGaN/GaN QWs were grown, in which indium composition was around 22 at.%. After that, 180 nm p-type AlGaN layer and 10 nm p^+^-type GaN capping layer were grown in sequence. The average thickness of InGaN wells and GaN barriers were 2.5 and 15 nm, respectively.

Temperature-dependent SSPL spectra and TRPL spectra were measured by using a Zolix-750 PL system equipped with a 30-mW He-Cd laser at 325 nm and a 10-mW pulsed laser at 377 nm as the excitation sources. PL decays were recorded by a time-correlated single-photon counting system at the temperature range from 10 to 300 K.

## Results and Discussion

**Fig. 2:**
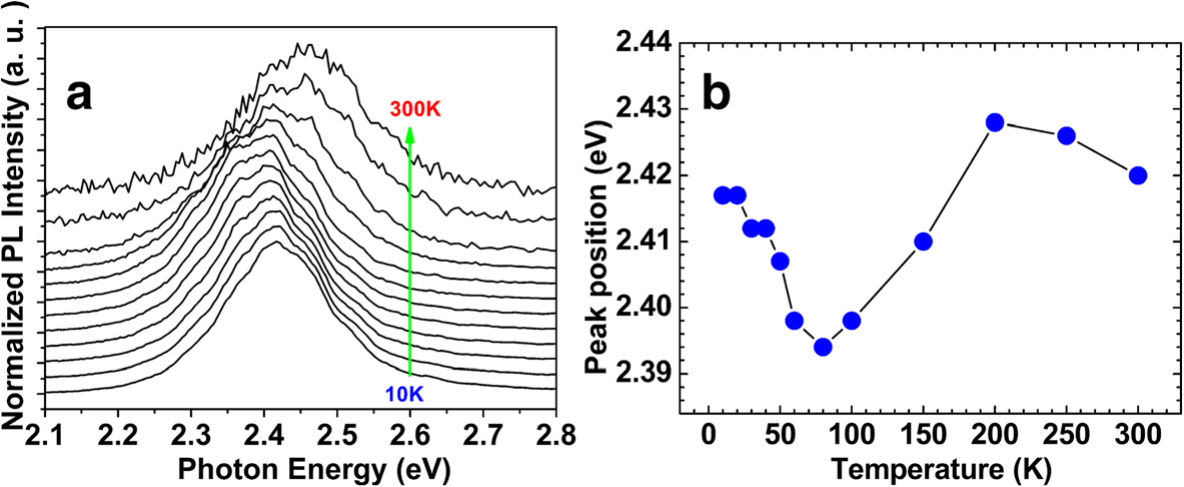
**a** Normalized temperature-dependent SSPL spectra showing the non-monotonic shift of emission peak position in the *green* MQW LED sample. **b** Detailed indication of the peak position shift

In order to understand the nature of microscopic mechanism of green emission from InGaN/GaN MQWs, we first measured the temperature-dependent SSPL spectra from 10 to 300 K, as shown in Fig. 2a. The emission peak position shifts non-monotonically with increasing temperature. Detailed illustration of the peak position shift was shown in Fig. 2b. With the temperature increasing from 10 to 70 K, the emission peak redshifts about 23 meV, much larger than the expected band-gap shrinkage of 4 meV over this temperature range [22]. Then, the emission peak blueshifts from 70 to 200 K. After the temperature further increasing above 200 K, the emission peak redshifts again. These anomalous “S-shaped” behaviors (Fig. 2b) have been explained by Cho and Feng et al. [6, 23–25], in which the basic assumption was that carrier localization at traps, originating from imperfections in InGaN layers, was the dominant pathway to give photons in InGaN active layers. Therefore, this phenomenon of “S-shaped” evolution of InGaN-related emission peak indicates that exciton localization remains the major origin of green emission from InGaN/GaN MQWs.

**Fig. 3:**
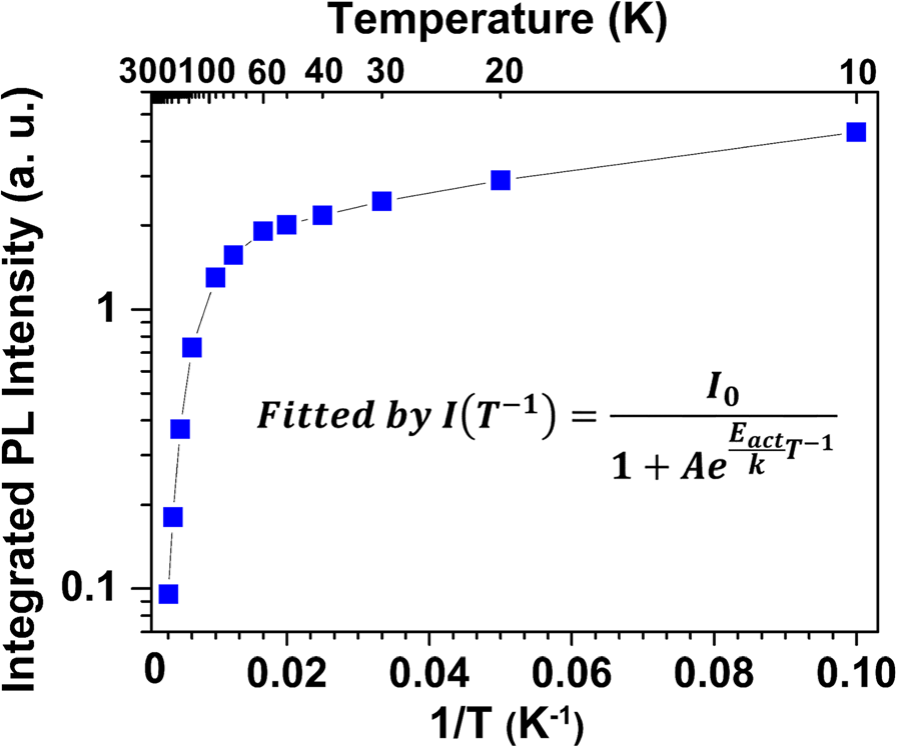
Temperature dependence of integrated PL intensity for the green MQW LED. An activation energy of 70 meV is obtained from Arrhenius plots

Figure 3 shows the corresponding evolution of integrated PL intensities of the green emission from InGaN/GaN MQWs with increasing temperature over the investigated range. It is found that the PL intensities decrease strongly with increasing temperature from 60 to 300 K, resulting from thermal quenching of PL intensities that is attributed to phonon-assisted non-radiative recombination. These intensities were fitted well with the Arrhenius equation (shown in Fig. 3). The obtained activation energy is about 70 meV, which is much less than the bandgap difference between well and barrier. That indicates that thermal quenching of the green emission is related to the dislocation of localized excitons, rather than thermal activation of electrons and/or holes from the InGaN wells into the GaN barriers. The external quantum efficiency at 300 K extracted from *I*(300*K*)/*I*_0_ was calculated as ~2%, which was obviously lower than that from blue MQW LEDs. This may be related to higher degree of non-radiative recombination centers existing in this green sample. Furthermore, the relative PL efficiencies for each emission photon energy can be evaluated as *η*_PL_(*T*, hν) = *I*(*T*, hν)/*I*_0_(hν). As shown in Fig. 2a, the PL peak continuously redshifts with similar peak shape at temperature range from 10 to 70 K. Based upon this, it is easy to estimate that *η*_PL_(*T*, hν) at low-energy side is higher than the one at high-energy side for each certain *T*. The maximum of *η*_PL_(*T*, hν) locates at low-energy side and redshifts following the growth of *T*. At the temperature range beyond 70 K, the PL peak tends to blueshift towards the position for 10 K, which makes the variety of *η*_PL_(*T*, hν) with *hv* at certain *T* become smaller.

**Fig. 4:**
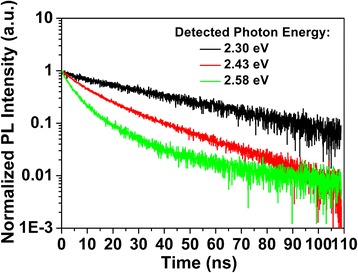
PL decay curves with different detected photon energy of the *green* MQW LED sample at 10 K

To shed light on the dynamic mechanism of localized excitons further, TRPL spectra of this green MQW LED sample were investigated. Figure 4 shows three typical decay curves of the PL intensities from different energy of the green PL band at 10 K, at which the influence of thermally activated non-radiative recombination was mostly excluded. As can be seen in the figure, the decay curves show single-exponential behaviors at low-energy region (2.30 eV) but deviate from single-exponential decay at high-energy region (2.58 eV). As our previous work [26], this phenomenon indicates that multiple PL pathways may exist in the high-energy region of PL.

**Fig. 5:**
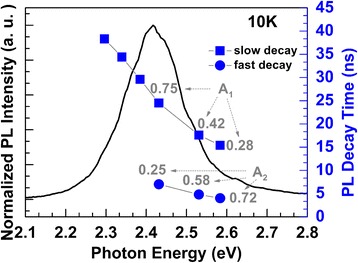
Photon energy dependence of PL decay times guiding by PL peak. The slow decay lifetime and fast decay lifetime were derived from bi-exponential decay function

PL decay curves of high-energy region were fitted by bi-exponential decay function (Eq. (1)), where two decay time were obtained [26],

1 $$ \frac{I(t)}{I_0}={A}_1{e}^{-\frac{t}{\tau_1}}+{A}_2{e}^{-\frac{t}{\tau_2}} $$

where *I*_0_ represents the PL intensity at *t* = 0, *τ*_1_ and *τ*_2_ represent the slow decay lifetime and the fast decay lifetime, respectively. *A*_1_ and *A*_2_ are related to the initial PL intensities of slow and fast decay process. Based on this model, the PL decay curves were split into two exponential decays. The obtained decay lifetimes at different photon energy were shown in Fig. 5, guided by the broad SSPL peak. The origins of slow PL process and fast PL process can be assigned to local compositional fluctuations of indium and thickness variation of InGaN layers, respectively [26]. It can be seen that the values of PL lifetime increase with decreasing photon energy for both fast and slow decays, which is ascribed to the energy transfer from a higher localized energy state to lower one. This is a characteristic of the localized system, where the decays of excitons consist of both radiative recombination and the transfer process to tail states. The depth of localization can be evaluated by assuming the exponential distribution of the density of tail states and by fitting the photon energy dependence of the *τ*_PL_ values using the following equation [27]:

2 $$ {\tau}_{\mathrm{PL}}={\tau}_{\mathrm{rad}}/\left[1+{e}^{\left( E-{E}_{\mathrm{me}}\right)/{E}_0}\right] $$

in which *τ*_rad_ represents the radiative recombination lifetime for free-exciton recombination in perfect InGaN single crystals; *E*_me_ is the energy value similar to the mobility edge, which means that an energy level higher than *E*_me_ is considered related to free state as well as an energy level lower than *E*_me_ is considered related to localized state. This value can be used to estimate the optical absorption edge of imperfect crystals containing localized tail states; *E*_0_ represents the depth of localization. Here, the obtained *E*_me_ for both fast and slow decay are the same as ~2.6 eV. If compared to the SSPL peak in Fig. 5, it indicates that the energy levels related to both kinds of recombination are totally below the mobility edge, so they are all ascribable to localized-state recombination; the obtained *τ*_rad_ is ~4 and ~40 ns for fast and slow decay, respectively, and *E*_0_ is ~20 meV for fast decay and ~65 meV for slow decay, which agrees well with the activation energy value obtained above.

It is also worth noted that the prefactors *A*_1_ and *A*_2_, associated with the ratio of fast and slow decay, were found various for different photon energy. The fraction of fast decay decreases with decreasing photon energy from 0.72 at 2.58 eV photon energy to 0.25 at 2.43 eV photon energy. For the emission energy lower than 2.43 eV, *A*_2_ is too small that only single-exponential decay fitting was used with *A*_1_ kept at 1. This phenomenon implies that fast decay is dominant at high-energy region of the emission as well as the slow decay is dominant at low-energy region.

**Fig. 6:**
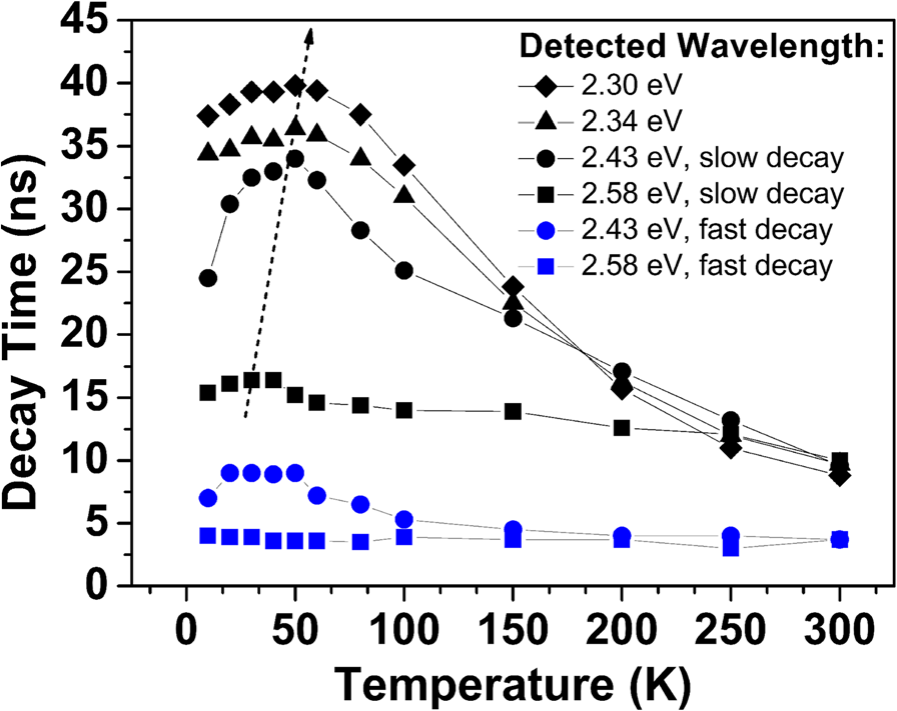
Temperature dependence of the decay times derived from bi-exponential decay function

Figure 6 shows the temperature dependence of the obtained decay lifetimes. From 60 to 300 K, all the decay lifetimes decrease with increasing the temperature, indicating the domination of non-radiative recombination in this range. In the range of 10 to 60 K, the decay lifetimes increase with increasing temperature. This is the evidence that the recombination occurred in certain localized states instead of some free states because free carrier recombination lifetime would be independent to temperature.

According to the models of Minsky et al. [28] and Chichibu et al. [29], in MQW system, there is the relation $1/{\tau}_{\mathrm{PL}}=1/{\tau}_{\mathrm{rad}}^{\hbox{'}}+1/{\tau}_{\mathrm{nr}}$, in which the radiative recombination lifetime ${\tau}_{\mathrm{rad}}^{\hbox{'}}$ contains both radiative free-exciton recombination and radiative recombination process through localized state, so it actually equals *τ*_PL_ in Eq. (2) at 10 K. In the case of InGaN wells, radiative free-exciton recombination lifetime *τ*_radf_ is much longer than the lifetime *τ*_L_ for radiative recombination through localized state; furthermore, *τ*_L_ is dominant by *τ*_loc_, the lifetime that free excitons are trapped by localized states, so ${\tau}_{\mathrm{rad}}^{\hbox{'}}\approx {\tau}_{\mathrm{loc}}$. The non-radiative lifetime *τ*_nr_ and the lifetime *τ*_loc_ can be deduced from abovementioned decay lifetimes with the combination of PL efficiency *η*_PL_(*T*) results. Qualitatively, the decline of PL lifetime at high-temperature range is dominant by the increase of non-radiative recombination rate, while the rise of PL lifetime at low-temperature range is dominant by the decrease of localization rate. Therefore, the temperature value is associated with the maximum of lifetime, which is an essential factor for evaluating the competition of radiative/non-radiative recombination processes. It can be seen from Fig. 6 that this point of maximum lifetime of slow decay shifts slightly to the high temperature when the detected phonon energy decreases from 2.58 to 2.30 eV. This indicates that the low-energy side of green PL peak, related to deeper localized states, is suppressed less by non-radiative recombination. This also accords the above *η*_PL_(*T*, hν) results, as *η*_PL_(*T*, hν) at low-energy side is higher than the one at high-energy side, and the maximum redshifts with increasing temperature. At the temperature range beyond 70 K, non-radiative delocalization process tends to dominate both fast and slow decay. The feature of different decay process becomes indistinguishable.

**Fig. 7:**
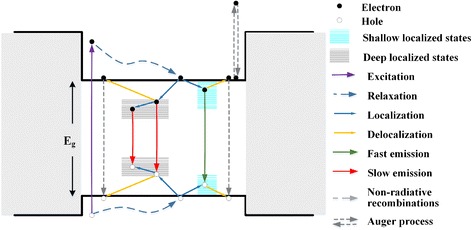
Schematics of the PL pathways in the green MQW LED

The schematic picture of the PL process in this green MQW LED sample has been done to illustrate the above measured results systematically. As seen in Fig. 7, the electrons in the valence band are pumped onto conduction band to generate excited carriers that far beyond the bandgap of InGaN well by absorbing the incident UV photons, and then parts of excited carriers relax to the states near the bandgap edge by releasing excess energy as heat. Theoretically, the radiative recombination rate of free excited carriers (free electron-hole pairs) in InGaN well layers is low because of the high density of dislocation in InGaN/GaN structure working as non-radiative trapping centers. Furthermore, the InGaN/GaN MQWs are grown on polar c-plane sapphire substrate, so a strain-induced built-in electric filed exists in the well layers. This is called quantum-confined Stark effect (QCSE). Despite some reported that polarization had positive effect to efficiencies of AlGaN LEDs or blue InGaN/GaN LEDs, for example increasing hole doping [30] or improving carrier tunneling [31], QCSE have been prove to be negative to InGaN/GaN LEDs with longer emission wavelength. That is because with increasing indium fraction, the InGaN/GaN lattice mismatch and strain become stronger, and the built-in field from QCSE will separate the different carriers in space, which will reduce the free carrier recombination rate in high degree [13, 32]. Fortunately, the imperfections of MQW structure, such as fluctuations of indium component inside well layers (which form deep states) and fluctuations of well thickness (which form shallow states) are available to capture free excitons or free electron-hole pairs to form localized excitons, preventing them from reaching the non-radiative dislocations. The recombination of localized excitons dominates the emission of InGaN wells, which has much higher recombination rate and shorter lifetime than free carriers. Meanwhile, the types of localization center are various as well as their localization depths, which leads to more complex dynamics of PL mechanism at different temperature. For example, the measured PL decay curves may deviate from single-exponential decay. It is worth noted that neither shallow nor deep localized states are located in single energy level but have certain distribution with a broad energy range within InGaN bandgap. The localization processes in different types of localization centers are independent to each other forming different PL pathways, but jumping may occur between states of one single type. For example, one exciton trapped by a localized state may jump to a deeper one with same type. This process leads to band-tail-like dynamic properties. The localized excitons are also possible to jump out of the localized states before radiative recombination by achieving the activation energy, that is, delocalize and become free excitons, then recombine through non-radiative pathways like Auger process. This jumping-out process strongly depends on the depth of the localized states. It will be harder for excitons to jump out from a deep trap because high temperature will be needed.

## Conclusions

In summary, temperature-dependent SSPL and TRPL spectra were studied for the green emission from InGaN/GaN MQW LED structure. S-shaped behavior of the SSPL peak position with increasing temperature was shown to be related to exciton localization. Two-step PL decay process was found in the high-energy region of the green PL emission band and degenerated to single-exponential decay toward low-energy region. This phenomenon was ascribed to two types of localized states contributed independently to the broad green PL emission band, and their state distributions were different and only had some overlap at high-energy region of the green emission. The deep localized states showed better resistance to the competitive non-radiative process than shallow states because the higher activation energy is needed for the delocalization process. These results showed a clear picture of excitation-emission process of the green MQW LED structure with broad emission band. Based on these results, some strategies for further improving the device efficiency can be proposed, such as inserting buffer layer or using patterned substrates, for purpose of releasing strain and reducing dislocation density on InGaN/GaN interfaces, which will decrease non-radiative recombination rate for fast PL process or intentional introducing In-rich clusters into well layers to increase the fraction of slow PL process with higher emission efficiency.

## Abbreviations

IQE: Internal quantum efficiency
LED: Light-emitting diode
MQW: Multiple quantum well
PL: Photoluminescence
QCSE: Quantum-confined Stark effect
SS: Steady state

## References

[1] Zeng F, Zhu L, Liu W, Li X, Liu W, Chen B-J (2016). Carrier localization and phonon-assisted hopping effects in semipolar InGaN/GaN light-emitting dioses grown by selective area epitaxy. J Alloys Compd.

[2] Il-Kyu P, Seong-Ju P (2011). Green gap spectral range light-emitting diodes with self-assembled InGaN quantum dots formed by enhanced phase separation. Appl. Phys. Express.

[3] Deng Z, Jiang Y, Ma Z, Wang W, Jia H, Zhou J (2013). A novel wavelength-adjusting method in InGaN-based light-emitting diodes. Sci Rep.

[4] Langer T, Kruse A, Ketzer FA, Schwiegel A, Hoffmann L, Jönen H (2011). Origin of the “green gap”: Increasing nonradiative recombination in indium-rich GaInN/GaN quantum well structures. Phys. Status Solidi C.

[5] De S, Layek A, Bhattacharya S, Kumar Das D, Kadir A, Bhattacharya A (2012). Quantum-confined stark effect in localized luminescent centers within InGaN/GaN quantum-well based light emitting diodes. Appl Phys Lett.

[6] Feng S-W, Cheng Y-C, Chung Y-Y, Yang CC, Lin Y-S, Hsu C (2002). Impact of localized states on the recombination dynamics in InGaN/GaN quantum well structures. J Appl Phys.

[7] Sun X, Li D, Song H, Chen Y, Jiang H, Miao G (2012). Short-wavelength light beam in situ monitoring growth of InGaN/GaN green LEDs by MOCVD. Nanoscale Res Lett.

[8] Yao HH, Lu TC, Huang GS, Chen CY, Liang WD, Kuo HC et al (2006) InGaN self-assembled quantum dots grown by metal–organic chemical vapour deposition with growth interruption. Nanotechnology 17:1713–6 10.1088/0957-4484/17/6/028 26558582

[9] Auf der Maur M, Pecchia A, Penazzi G, Rodrigues W, Di Carlo A (2016). Efficiency drop in green InGaN/GaN light emitting diodes: the role of random alloy fluctuations. Phys Rev Lett.

[10] Jeong H, Jeong HJ, Oh HM, Hong C-H, Suh E-K, Lerondel G (2015). Carrier localization in In-rich InGaN/GaN multiple quantum wells for green light-emitting diodes. Sci Rep.

[11] Sousa MA, Esteves TC, Sedrine NB, Rodrigues J, Lourenço MB, Redondo-Cubero A (2015). Luminescence studies on green emitting InGaN/GaN MQWs implanted with nitrogen. Sci Rep.

[12] Li Z, Kang J, Wei Wang B, Li H, Hsiang Weng Y, Lee Y-C (2014). Two distinct carrier localization in green light-emitting diodes with InGaN/GaN multiple quantum wells. J Appl Phys.

[13] Feng ZC, Zhu LH, Kuo TW, Wu CY, Tsai HL, Liu BL (2013). Optical and structural studies of dual wavelength InGaN/GaN tunnel-injection light emitting diodes grown by metalorganic chemical vapor deposition. Thin Solid Films.

[14] O'Donnell K, Martin R, Middleton P (1999). Origin of luminescence from InGaN diodes. Phys Rev Lett.

[15] Yang TJ, Shivaraman R, Speck JS, Wu YR (2014). The influence of random indium alloy fluctuations in indium gallium nitride quantum wells on the device behavior. J Appl Phys.

[16] Park I-K, Kwon M-K, Kim J-O, Seo S-B, Kim J-Y, Lim J-H et al (2007) Green light-emitting diodes with self-assembled In-rich InGaN quantum Dots. Appl Phys Lett 91(13):133105

[17] Yang Y, Ma P, Wei X, Yan D, Wang Y, Zeng Y (2014). Design strategies for enhancing carrier localization in InGaN-based light-emitting diodes. J Lumin.

[18] Zhang M, Bhattacharya P, Guo W (2010) InGaN/GaN self-organized quantum dot green light emitting diodes with reduced efficiency droop. Appl Phys Lett 97(1):011103

[19] Liu W, Zhao DG, Jiang DS, Chen P, Liu ZS, Zhu JJ (2015). Localization effect in green light emitting InGaN/GaN multiple quantum wells with varying well thickness. J Alloys Compd.

[20] Yamamoto S, Zhao Y, Pan C-C, Chung RB, Fujito K, Sonoda J et al (2010) High-efficiency single-quantum-well green and yellow-green light-emitting diodes on semipolar (2021) GaN substrates. Appl Phys Express 3(12):122102

[21] Feezell DF, Schmidt MC, Den Baars SP, Nakamura S (2009) Development of nonpolar and semipolar InGaN/GaN visible light-emitting diodes. MRS Bull 34(5):318–23

[22] Chua SJ, Soh CB, Liu W, Teng JH, Ang SS, Teo SL (2008) Quantum dots excited InGaN/GaN phosphor-free white LEDs. Phys Status Solidi C 5(6):2189–91

[23] Cho YH, Gainer GH, Fischer AJ, Song JJ, Keller S, Mishra UK (1998). “S-shaped” temperature-dependent emission shift and carrier dynamics in InGaN/GaN multiple quantum wells. Appl Phys Lett.

[24] Liu T, Jiao S, Wang D, Gao S, Yang T, Liang H (2015). Radiative recombination mechanism of carriers in InGaN/AlInGaN multiple quantum wells with varying aluminum content. J Alloys Compd.

[25] Li Q, Xu SJ, Cheng WC, Xie MH, Tong SY, Che CM (2001). Thermal redistribution of localized excitons and its effect on the luminescence band in InGaN ternary alloys. Appl Phys Lett.

[26] Lin T, Qiu ZR, Yang J-R, Ding LW, Gao Y, Feng ZC (2016). Investigation of photoluminescence dynamics in InGaN/GaN multiple quantum wells. Mater Lett.

[27] Yang F, Wilkinson M, Austin EJ, O'Donnell KP (1994). Origin of the stokes shift: a geometrical model of exciton spectra in 2D semiconductors. Phys Rev Lett.

[28] Minsky MS, Watanabe S, Yamada N (2002). Radiative and non-radiative lifetimes in GaInN/GaN multiquantum wells. J Appl Phys.

[29] Chichibu S, Onuma T, Sota T, DenBaars SP, Nakamura S, Kitamura T (2003). Influence of InN mole fraction on the recombination processes of localized excitons in strained cubic InxGa1-xN/GaN multiple quantum wells. J Appl Phys.

[30] Li S, Zhang T, Wu J, Yang Y, Wang Z, Wu Z (2013). Polarization induced hole doping in graded AlxGa1 − xN (x = 0.7 ∼ 1) layer grown by molecular beam epitaxy. Appl Phys Lett.

[31] Zhang Z-H, Tiam Tan S, Kyaw Z, Ji Y, Liu W, Ju Z (2013). InGaN/GaN light-emitting diode with a polarization tunnel junction. Appl Phys Lett.

[32] Liu L, Wang L, Liu N, Yang W, Li D, Chen W (2012). Investigation of the light emission properties and carrier dynamics in dual-wavelength InGaN/GaN multiple-quantum well light emitting diodes. J Appl Phys.

